# Mortality comparison in acute myocardial infarction patients according to presence versus absence of traditional modifiable cardiovascular risk factors

**DOI:** 10.6026/973206300220917

**Published:** 2026-02-28

**Authors:** Jitendra Kanjolia, Ashis Tiwari, Sukh Dayal Kumhar, Praveen Kumar Tagore, Shivani Parihar, Abha Bardiya

**Affiliations:** 1 Department of General Medicine, Government Medical college, Datia, Madhya Pradesh, India; 2 Department of Pathology, Chirayu Medical College, Bhopal, Madhya Pradesh, India; 3 Department of Anaesthesiology, Shyam Shah Medical College, Rewa, Madhya Pradesh, India

**Keywords:** Acute myocardial infarction (AMI), SMuRFs, mortality, risk factor paradox, cardiogenic shock

## Abstract

Standard modifiable cardiovascular risk factors (SMuRFs) like hypertension, diabetes, dyslipidemia and smoking strongly predict AMI, yet many patients lack these. This retrospective cohort study analyzed 580 consecutive Type 1 AMI admissions, stratifying into SMuRF-less (0 factors) versus those with ≥1 SMuRF. SMuRF-less patients showed significantly higher in-hospital mortality (10.9% vs. 5.2%, p=0.03). They also exhibited increased cardiogenic shock frequency compared to the SMuRF-present group. SMuRF-less AMI advances understanding by revealing paradoxical mortality risk, necessitating expanded screening beyond traditional factors.

## Background:

The most devastating symptom of cardiovascular disease, which is the leading global killer, is acute myocardial infarction (AMI). Traditional modifiable cardiovascular risk factors (SMuRFs) such as hypertension, diabetes mellitus, dyslipidaemia and cigarette smoking have been the foundation for risk assessment and cardiovascular disease prevention for many years. These four elements were first highlighted in the Framingham Heart Study and subsequently validated on a global scale by the INTERHEART trial, which found that they accounted for over 90% of the population-attributable risk of myocardial infarction [1]. This, therefore, means that clinical suspicion of ACS is usually lower in individuals who do not present with these classic signs. Nevertheless, according to the recent epidemiological data, a significant percentage of patients with AMI (10-27% of them) do not have any of these typical risk factors [2]. This subgroup that is becoming more and more called SMuRF- less patients posed a distinct clinical challenge. Although it would be intuitively presumed that lack of the presence of systemic metabolic disease and vascular risk factors would provide a survival benefit, new data seems to indicate the presence of a risk factor paradox [3]. A number of large-scale registries have stated that SMuRF- fewer patients tend to have poorer short-term outcomes than those patients who have numerous risk factors [4]. The mechanisms behind this paradox are still unclear. Hypotheses go from absence of ischemic preconditioning (in which chronic atherosclerosis facilitates the development of collateral vessels to protect against acute occlusion) to the existence of unidentified nontraditional risk factors, e.g. high levels of Lipoprotein(a), clonal hematopoiesis of indeterminate potential (CHIP) or hypercoagulable states [5]. Moreover, the lack of conventional risk factors is feared to result in delays associated with reperfusion therapy and diagnosis because of the decreased clinical suspicion on arrival at the emergency department [6]. Although there is an increasing awareness of this phenotype, there is still inconsistent literature comparing in-hospital mortality and procedural outcomes in tertiary care hospitals with some studies demonstrating no difference and others demonstrating excess mortality. The current state of knowledge has a critical research gap regarding the understanding of whether the enhanced mortality is facilitated by clinical presentation (e.g., higher rates with cardiac arrest) or differences in management [7]. Therefore, it is of interest to compare the clinical manifestations, angiographic findings and in-hospital mortality rates of acute myocardial infarction (AMI) patients with and without the commonly identified modifiable cardiovascular risk factors.

## Materials and Methods:

## Study design and setting:

Located in the cardiology department of Government medical College Datia, Madhya Pradesh, India, this research was a retrospective observational cohort study with a single location. Start date of the research was August 2025 and end date was October, 2025.

## Sample size and population:

A total of 580 consecutive adult patients (≥ 18 years) admitted with a primary diagnosis of Type 1 Acute Myocardial Infarction (both STEMI and NSTEMI) were included.

## Inclusion criteria:

[1] Diagnosis of AMI defined by the Fourth Universal Definition of Myocardial Infarction (rise/fall of cardiac troponin with at least one value >99th percentile URL, plus symptoms of ischemia or ECG changes).

[2] Underwent coronary angiography during the index hospitalization.

## Exclusion criteria:

[1] Type 2 MI (secondary to supply/demand mismatch).

[2] Prior history of coronary artery disease (prior MI, PCI, or CABG), as this alters the risk factor profile and collateralization.

[3] Incomplete data regarding baseline risk factors.

[4] Death prior to hospital arrival.

## Group definitions:

Patients were stratified into two cohorts based on the presence of Standard Modifiable Cardiovascular Risk Factors (SMuRFs):

[1] Group A (SMuRF-less): Patients with zero standard risk factors.

[2] Group B (SMuRF-positive): Patients with 1 standard risk factor.

## The SMuRFs were defined as:

[1] Hypertension: Previous diagnosis, current use of antihypertensive medication, or admission BP >140/90 mmHg on two occasions (excluding the acute pain phase).

[2] Diabetes mellitus: Previous diagnosis, use of insulin/oral hypoglycemics, or HbA1c 6.5%.

[3] Dyslipidaemia: Previous diagnosis, use of lipid-lowering agents, or Total Cholesterol >200 mg/dL / LDL >130 mg/dL.

[4] Smoking: Current smoker or cessation within the last 12 months.

## Data collection:

Data were extracted from electronic medical records (EMR). Variables included:

[1] Demographics: Age, sex, BMI.

[2] Clinical Presentation: Type of MI (STEMI versus NSTEMI), Killip class, presence of cardiac arrest, symptom-to-door time and door-to-balloon time.

[3] Angiographic Data: Number of vessels involved (Single versus Multi-vessel disease), culprit lesion location and TIMI flow grade.

[4] Outcomes: The primary outcome was in-hospital all-cause mortality. Secondary outcomes included cardiogenic shock requiring vasopressors/mechanical support and acute left ventricular failure.

## Statistical analysis:

Statisticians at IBM Corp. in Armonk, NY, used SPSS 26.0 to compile and analyze the data. The mean ± standard deviation (SD) was used to describe continuous variables and the Student's t-test or Mann-Whitney U test, based on normality, was used for comparisons. The Chi-square test or Fisher's exact test were used for comparing categorical variables, which were presented as percentages and frequencies. After taking age, sex and MI type (STEMI/NSTEMI) into account, a multivariate logistic regression analysis was run to see whether "SMuRF-less" status remained a significant predictor of death after excluding any confounding variables. It was deemed statistically significant if the p-value was less than 0.05.

## Results:

Of the 580 patients included in the study, 82 patients (14.1%) were classified as SMuRF-less (Group A), while 498 patients (85.9%) had at least one traditional risk factor (Group B). A look at the demographic profile showed some interesting variations. As compared to the SMuRF-positive group, the SMuRF-less patients were noticeably younger (Mean age 58.4 ± 12.1 years versus 64.2 ± 11.5 years; p < 0.01) and had a lower average Body Mass Index (BMI). More men (78.0% vs.66.5%) were in the group that did not have SMuRF. The study's protocol indicated that there was no history of smoking, hypertension, diabetes or dyslipidaemia in (Group A) (Table 1). A higher likelihood of Single-Vessel Disease (SVD) was seen in angiographic examination in individuals without SMuRF compared to the risk factor group, which had substantial Multi-Vessel Disease (MVD) (65.9% versus 38.2%, p < 0.001). For the group without SMuRF, the culprit vascular most often was the Left Anterior Descending (LAD) artery. Total occlusion rates (TIMI 0 flow) were high in the SMuRF-less group even though they had less complicated atherosclerosis (a smaller load of chronic plaque) at baseline. Door-to-balloon times were statistically similar between groups (78 ± 25 min versus 74 ± 22 min, p=0.45), suggesting no significant delay in in-hospital processing once the diagnosis was made (Table 2). With a p-value of just 0.03 between the SMuRF-positive and SMuRF-negative groups, the main outcome of in-hospital all-cause death was much higher in the former. Furthermore, the rate of cardiogenic shock was approximately two times higher in the group that did not have SMuRF (15.9% versus 8.4%). The multivariate logistic regression model found that SMuRF-less status was still a significant predictor of in-hospital mortality (Odds Ratio 2.15, 95% CI 1.05-4.42, p = 0.036) even after controlling for age, sex and STEMI presentation (Table 3).

## Discussion:

The key finding of this study is that the in-hospital mortality rate for patients with acute myocardial infarction who do not have standard modifiable cardiovascular risk factors (SMuRFs) is much higher than that of patients who do have conventional risk factors. Despite being younger (mean age 58 versus 64) and having a less advanced burden of angiographic atherosclerosis (mainly single-vessel disease), the SMuRF-less group had a higher death rate than the SMuRF-positive one, albeit the difference was more than double (10.9 versus 5.2). These findings support the presence of the so-called risk factor paradox and the most recent data on a large scale are offered by the SWEDEHEART registry and works by Vernon *et al.* [8]. A number of pathophysiological processes could be used to account for these counter-intuitive results. The first of all is the lack of ischemic preconditioning. The diffuse atherosclerosis is likely to develop over the years in patients with long-standing diabetes, hypertension and dyslipidaemia. This is a persistent ischemia that provokes the growth of collateral circulation. The collateral flow has the potential to rescue the myocardium when there is an acute occlusion in these patients, thus reducing the size of the infarcts [9]. Conversely, SMuRF-depleted patients in our study were more likely to present with single- vessel disease and a high burden of thrombus indicating an embolic, acute or extremely thrombotic event within a naive coronary artery. In the absence of collateral protection, the acute occlusion results in a rapid and massive loss of viable myocardium, triggering cardiogenic shock, which was much more common in our SMuRF less cohort (15.9%) [10]. Secondly, the etiology of thrombosis in patients without SMuRF could be different. Whereas the traditional MI is characterized by plaque rupture, SMuRF-less MI can be caused more often by plaque erosion or other endothelial dysfunction agents which are not traditional [11]. These may be driven by high concentration of inflammatory markers, genetic predisposition like high Lipoprotein (a) or by coagulopathies that cannot be detected. In particular, Lipoprotein (a) has been recognized as an independent risk causal factor not dependent on conventional lipids and common in younger healthy MI patients [12]. Its analysis did not always quantify Lp(a), which points to one of the possible aspects of routine screening. Of special concern is the increased rate of out-of-hospital cardiac arrest (11.0% versus 5.0% in the SMuRF- less group). This is an indication that initial presentation of CAD among these people is most of the time disastrous. This differs with traditional triage heuristics applied in emergency medicine and primary care clinically. A patient with no history of diabetes or hypertension and with a healthy appearance and a complaint of chest pains could be considered to be potentially better placed to die in the immediate future in case of an MI than a patient with a number of comorbidities [13]. What is more, the secondary prevention is not easy in this group. They do not have hypertension or dyslipidemia (by standard definitions) and thus they might be ineligible to reimbursements on some of the therapies, or might be less adherent to statins and ACE inhibitors since they think they are low risk after the acute event has been experienced [14]. Nevertheless, our multivariate analysis can prove that SMuRF-less status is an independent mortality predictor (OR 2.15) and requires aggressive guideline-based medical treatment independent of initial lipid or BP rates. Our tendency to have SMuRF-less patients (14.1) is in line with the world average of 15-20% of patients with SMuRF [15]. Nevertheless, we have slightly more mortality rates than other registry data, probably because of the tertiary nature of our center receiving critical transfers and high-risk cases of STEMI. Notably, our results are in conflict with previous assumptions that young patients with no risk factors have an insidious course; instead, it is a risk group with a high propensity to an acute hemodynamic crisis [16, 17]. This article has certain weaknesses due to its retrospective nature and focus on in-hospital outcome. We were not able to evaluate long- term survival and heart failure development after discharge. Also, we used EMR documentation of risk factors; there might be patients with unidentified pre-diabetes or untreated hypertension, but we used admission measurements to control it. Lastly, no non-traditional biomarkers were available to us (e.g. homocysteine, Factor V Leiden, Lp (a) and therefore we were not able to determine the exact etiology in Group A.

## Conclusion:

The SMuRF-less status is not to be considered the sign of good prognosis, it reveals the high-risk phenotype with the sudden and widespread myocardial damage with no collateral circulation. Patients without the classic risk factors and severe coronary events require the clinician to be very suspicious and provide aggressive acute management. Future studies should work on screening the non-traditional biomarkers that cause atherosclerosis in this subgroup to come up with specific preventive strategies.

## Figures and Tables

**Table 1:** Baseline demographic and clinical characteristics

**Variable**	**SMuRF-less (n=82)**	**SMuRF-positive (n=498)**	**P-value**
Age (years), Mean ± SD	58.4 ± 12.1	64.2 ± 11.5	0.002
Male Sex, n (%)	64 (78.0)	331 (66.5)	0.04
BMI (kg/m^2^), Mean ± SD	24.8 ± 3.2	28.5 ± 4.8	<0.001
STEMI Presentation, n (%)	58 (70.7)	260 (52.2)	0.003
Out-of-Hospital Cardiac Arrest, n (%)	9 (11.0)	25 (5.0)	0.03
LVEF < 40% on admission, n (%)	28 (34.1)	135 (27.1)	0.19

**Table 2:** Angiographic characteristics

**Variable**	**SMuRF-less (n=82)**	**SMuRF-positive (n=498)**	**P-value**
Single Vessel Disease, n (%)	54 (65.9)	190 (38.2)	<0.001
Multi-Vessel Disease, n (%)	28 (34.1)	308 (61.8)	<0.001
Culprit: LAD, n (%)	45 (54.9)	210 (42.2)	0.03
Pre-PCI TIMI Flow 0/1, n (%)	60 (73.2)	290 (58.2)	0.01
Thrombus Burden (High), n (%)	35 (42.7)	150 (30.1)	0.02

**Table 3:** In-hospital clinical outcomes

**Outcome**	**SMuRF-less (n=82)**	**SMuRF-positive (n=498)**	**P-value**
In-hospital Mortality, n (%)	9 (10.9)	26 (5.2)	0.03
Cardiogenic Shock, n (%)	13 (15.9)	42 (8.4)	0.04
New onset Atrial Fibrillation, n (%)	4 (4.9)	38 (7.6)	0.38
Major Bleeding, n (%)	2 (2.4)	18 (3.6)	0.72
